# Fertility and sexual dysfunction in young male cancer survivors

**DOI:** 10.1002/rmb2.12481

**Published:** 2022-08-06

**Authors:** Yasushi Yumura, Teppei Takeshima, Mitsuru Komeya, Shinnosuke Kuroda, Tomoki Saito, Jurii Karibe

**Affiliations:** ^1^ Department of Urology, Reproduction Center Yokohama City University, Medical Center Yokohama City Japan

**Keywords:** chemotherapy, ejaculation, erectile dysfunction, radiotherapy, spermatogenesis

## Abstract

**Background:**

Newly emerging serious post‐treatment complications among young male cancer survivors involve fertility and sexual function, preventing them from pursuing a normal family life.

**Methods:**

We studied and summarized published studies that assess the relationship between cancer treatments and reduced spermatogenesis or sexual dysfunction.

**Main findings:**

Infertility often occurs because of anticancer therapies that impair spermatogenesis. While some patients postremission functionally recover fertility, others experience a decreased sperm count and azoospermia. Fertility‐preserving modalities are currently being promoted worldwide to preserve spermatogenesis following cancer therapy. Patients who can ejaculate and have sperm in their semen should cryopreserve semen. However, for patients who have never ejaculated before puberty or in whom spermatogenesis has not been established, testis biopsy is performed to collect and preserve sperm or germ cells. Fertility preservation is gaining popularity and requires continuous information dissemination to oncologists and cancer treatment professionals. Furthermore, male sexual dysfunction predominantly involves erectile dysfunction and ejaculation disorder.

**Conclusion:**

Although preventive and therapeutic methods for these disorders have been established within urology, patients and medical professionals in other fields remain uninformed of these advances. Therefore, dissemination of information regarding fertility preservation techniques should be accelerated.

## Graphical abstract

Young male patients treated for cancer may experience decreased fertility and sexual function after treatment. This article described the causes of these failures and countermeasures.

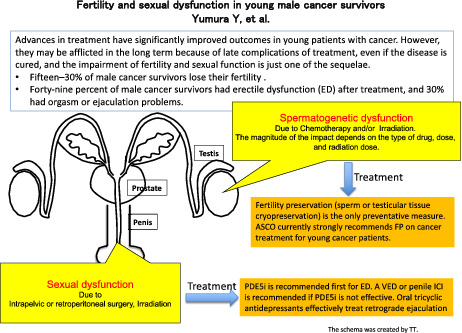

## 1 INTRODUCTION

Advances in treatment have significantly improved outcomes in young patients with cancer. The 3‐year survival rate of patients with advanced testicular cancer in Japan is 74.3%–94.7%, depending on the risk.^1^ The 5‐year survival rates for bone and soft tissue tumors were 71.8% for children and 73.1% for young people (15–29 years old).^2^ For blood malignancy, the 10‐year relative survival rate was 76.5% for those aged 0–14 years and 52.5% for those aged 15–29 years, although this included men and women. The 10‐year relative survival rates for malignant lymphoma were 88.6% and 73.4% for those aged 0–14 and 15–29 years, respectively, and those for brain tumors were 58% and 58.9% for those aged 0–14 and 15–29 years, respectively.^3^

Young cancer patients may be afflicted in the long term because of late complications of treatment, even if the disease is cured, and the impairment of fertility and sexual function is just one of the sequelae. Some patients may forgo marital or other intimate partnerships due to sexual dysfunction or infertility, and others may be forced to make major changes to their life plans. Benedict et al. conducted a survey of 43 men and women who survived cancer during their adolescence and young adult (AYA) generation about their fertility; half of the men reported uncertainty regarding their fertility status.^4^ In a study involving survivors of cancer treatment in the AYA generation, 48.8% had impaired sexual function and intimate relationships 1 year after treatment; even after 2 years, 70% of survivors continued to experience these effects.^5^

As the treatment outcomes of young cancer patients have improved, it is important to communicate information regarding novel treatment and prevention methods for these sequelae to provide them hope for the future.

## 2 DECREASED FERTILITY DUE TO CANCER TREATMENT

Disorders of spermatogenesis after chemotherapy or radiation therapy are among the most problematic sequelae of cancer treatment, and 15%–30% of male cancer survivors lose their fertility.^6^ Walsile‐Masker et al. conducted a questionnaire survey of 1622 male cancer survivors and 264 siblings on post‐cancer infertility; the incidence of infertility in the cancer survivor cohort was 46%, and that in siblings was 17.5% (relative risk (RR) = 2.34, 95% confidence interval [CI] 1.88–3.70, *p* < 0.001). Most causes of infertility are due to the gonadotoxic effects of chemotherapy or radiation therapy.^7^

### 2.1 Impact of cancer treatment on spermatogenesis

Spermatogonia are highly sensitive to the cytotoxic effects of radiation and anticancer drugs. In particular, agent sensitivity increases during differentiation.^8^ Later‐stage germ cells, on the other hand, are relatively resistant to cell‐killing effects. Therefore, although the number of spermatogonia decreases immediately after cancer treatment, spermatogenesis from later‐stage germ cells (spermatocytes and spermatids) continues. A study indicated that the sperm count did not change significantly immediately after the start of treatment but dropped sharply from 1/10 to 1/100 1–2 months after the start of treatment, and azoospermia may occur 12 weeks after treatment, depending on the drug and dosage.^9^ In the case of a drug with a low cytotoxic effect, the sperm count normalizes again about 12 weeks after the end of chemotherapy.

In contrast, in treatments with high cell‐killing effects, the pros and cons of sperm function recovery and the extent and time of recovery, depend on the number of stem cells remaining after treatment.^9^ The number of surviving spermatogonia depends on the type of drug, regimen, and dose. For example, alkylating agents are highly toxic to the testes, and with cyclophosphamide, azoospermia is prolonged at a total dose of 19 g/m^2^ for a single agent and 7.5 g/m^2^ or more for a combination of multiple agents.^10^ Ifosfamide increases the damage at total doses of 42 g/m^2^ and above.^11^ Cisplatin increases the risk of azoospermia at doses >400 mg/m^2^.^8^ Magelssen et al. administered cisplatin‐based chemotherapy to 170 germ cell tumor patients who had normozoospermia before treatment and investigated semen findings at least 1 year after treatment; 64% of patients had normozoospermia, 16% had oligozoospermia, and 20% showed azoospermia.^12^ In the case of radiation therapy, spermatocytes begin to decrease approximately 21 weeks after a single irradiation of 0.2–4 Gy, and differentiation into spermatocytes also decreases.^13^, ^14^ In general, the sperm count begins to decrease when the irradiation dose to the testes is 0.15 Gy or higher, and reversible azoospermia is exhibited at 0.35–0.5 Gy. Nadir with decreased sperm count occurs 4–6 months after the end of treatment and takes 10–18 months to recover fully. If the dose increases, the recovery time is extended, but the original sperm count may not be re‐established in cases undergoing irradiation of 1.2 Gy or more.^11^, ^15^ Only 15% of patients recover fertility with a single dose of 10 Gy,^9^ and 95% present with Sertoli cell‐only syndrome when the dose exceeds 16–18 Gy.^16^

Fractionated irradiation is effective for cancer treatment; however, although damage to the irradiated area is reduced, damage to the testes is significant. Cumulative doses to the testes above a total of 2.5 Gy can lead to irreversible azoospermia.^11^, ^15^ The combined damage of chemotherapy and radiation therapy is significant, with 83% of patients presenting with permanent azoospermia due to total body irradiation and cyclophosphamide administration prior to stem cell transplantation in leukemia.^17^

Based on these findings, the American Society of Clinical Oncology (ASCO) classifies the risks to sperm function in anticancer drugs and radiation therapy into four stages (Table 1).^18^ Treatments classified as high risk may prolong azoospermia, and it is advisable to preserve sperm before treatment.

**TABLE 1:** Effects of different anti‐tumor agents on sperm production: ASCO's fertility preservation guideline update^17^

High risk	Intermediate risk	Lower risk	Very low risk	Unknown
Any alkylating agent + total body irradiation	BEP >2–4 cycles Total cisplatin >400 mg/㎡ Total carboplatin >2 g/m^2^	Protocol containing nonalkylating agents ABVD,CHOP	Multi‐agent therapies containing vincristine	Monoclonal Antibodies
Any alkylating agent + pelvic or testicular radiation		Testicular radiation <0.2–0.7Gy	Radioactive iodine	Tyrosine kinase inhibitors
Total cyclophosphamide >7.5 g/m^2^		Antracycline + cytarabine	Testicular radiation (due to scatter) <0.2 Gy	
Testicular radiation >2.5 Gy in men >6 Gy in boys	Testicular radiation (due to scatter) 1‐6Gy			
Protocol containing procarbazine: MOPP >3 cycles BEACOPP >6 cycles				
Protocol containing temozolomide or BCNU +cranial radiation				
Total body irradiation (TBI)				
Cranial radiation >40Gy				

*Note*: High risk: prolonged/permanent azoospermia common after treatment; intermediate risk: prolonged/permanent azoospermia is not common after treatment but can occur; low risk: treatments typically cause only temporary damage to sperm production; very low risk: no effect on sperm production.

In recent years, cancer treatment with molecular target agents, such as tyrosine kinase inhibitors (TKIs), has been increasing. TKIs suppress cancer proliferation by targeting molecules present on cells and suppressing cell proliferation. Currently, the risk classification of the ASCO guidelines still states that the adverse effects on spermatogenesis are unknown.^18^ However, there are reports that TKI treatment may lead to a risk of decreased fertility; hence, data needs to be collected in the future.

### 2.2 Recovery of fertility after cancer treatment

As mentioned above, recovery of spermatogenesis after cancer treatment depends on the number of surviving spermatogonia.^9^ If spermatogonia do not remain after treatment with a highly cell‐lethal drug or high dose, azoospermia occurs, and even if spermatogonia remain, it may take several years for genesis to recover. Even in a moderate‐risk chemotherapy regimen, the time to recovery will be delayed if the dose and number of courses are high. Suzuki et al. investigated the time to recovery of spermatogenesis in 45 patients who received chemotherapy for testicular cancer (bleomycin, etoposide, cisplatin [BEP] therapy). Forty‐four patients recovered their spermatogenesis, but the time to recovery was extended as the number of cycles increased. No patients in the group who received more than 5–6 courses of BEP therapy recovered their spermatogenesis within 2 years.^19^ Martinez et al. reported that it takes approximately 1 year after adriamycin, bleomycin, vinblastine, dacarbazine (ABVD) therapy and 2 years after cyclophosphamide, hydroxydaunorubicin (adriamycin), oncovin (vincristine), prednisone/mechlorethamine, oncovin (vincristine), procarbazine, prednisone‐adriamycin, bleomycin, vinblastin (CHOP/MOPP‐ABV) therapy to recover to pretreatment sperm count levels in patients with malignant lymphoma.^20^ Similarly, sperm count can be recovered at low doses with radiation therapy, but it takes 7 months to recover after 1 Gy irradiation and 24 months after 6 Gy.^9^

Some young cancer survivors are concerned about sperm chromosomal abnormalities and DNA fragmentation, because this may increase the rate of malformation in their children. In the case of testicular cancer, two or more courses of BEP therapy increase the proportion of sperm with chromosomal aneuploidy; however, this effect diminishes after 24 months.^21^ In malignant lymphoma, the proportion of sperm with aneuploidy is higher than before treatment up to 3 months after treatment with ABVD and up to 12 months after treatment with CHOP but then decreases.^20^ Thomson et al. compared semen analyses and sperm DNA fragmentation rates of male cancer survivors who underwent cancer treatment in childhood with those of a normal control group. Eleven of the 33 cancer survivors had azoospermia, and the remaining patients also had decreased sperm counts, but the DNA fragmentation rate was not significantly different from that of the normal control group.^22^ Sperm DNA fragmentation was also measured before and after treatment in patients with testicular cancer who received chemotherapy and radiation therapy. It was demonstrated that the fragmentation rate increased 6 months after treatment, but there was no significant difference, and it returned to pretreatment levels in about 1 year.^23^

## 3 THE PRACTICE OF FERTILITY‐PRESERVING TREATMENT IN MALE

ASCO published its guidelines in 2006 considering the fertility crisis associated with cancer treatment. Sperm banking should be offered to postpubertal male cancer patients receiving cancer treatments.^18^, ^24^, ^25^ In Japan, guidelines were published by the Cancer Treatment Society in July 2017. The guidelines presuppose that cancer treatment is the priority but that oncologists should inform patients of reproductive age about the possibility of associated infertility. They should also evaluate the presence and timing of fertility preservation in collaboration with reproductive specialists.^26^

### 3.1 Cryopreservation of sperm or testicular tissue

**FIGURE 1:**
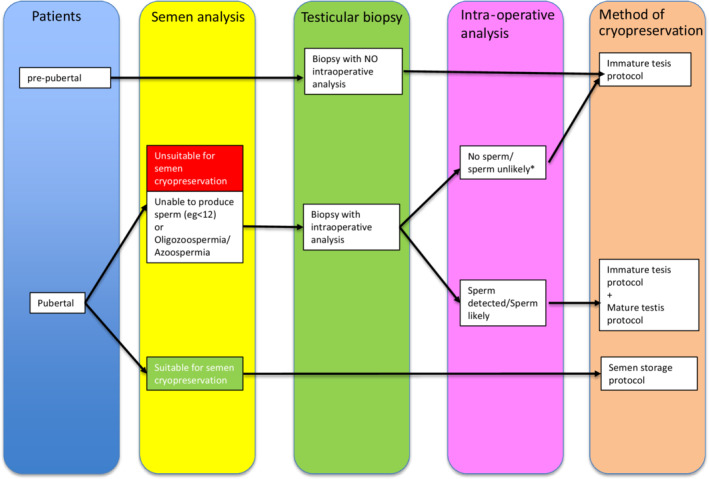
Algorithm developed by Picton et al. for cryopreservation of testicular tissue and sperm in prepubertal and pubertal patients, which should be used prior to high‐risk treatments that may lead to infertility.^26^ Pubertal patients are first tested for semen, and the storage protocol is performed if the semen contains enough storable sperm. Patients who are unable to produce sperm (including those who cannot ejaculate) or those with oligozoospermia or azoospermia undergo testicular biopsy (Onco‐TESE). Intraoperative analysis is performed on the tissues obtained through biopsy, and any sperm detected are stored (mature testis protocol). The immature testis protocol is a method of cryopreserving tissues that contain sperm‐like cells (spermatogonia to spermatids). Since there is a high possibility that there are no sperm in the tissue, cryopreservation is expected with the hope that future medical advances will enable the differentiation of sperm‐like cells (germ cells) into sperm cells. In prepubertal patients, the tissue is collected and cryopreserved on the premise that masturbation is not possible (immature testis protocol).

Cryopreservation of sperm or testicular tissue is the most prevalent fertility‐preserving treatment modality for men. The European Society of Human Reproduction and Embryology Task Force designed a cryopreservation algorithm for the preservation of sperm and testicular tissue in prepubertal and adolescent men at risk of loss of fertility (Figure 1).^27^ Sperm cryopreservation is performed in men after puberty if ejaculation is possible. If the patient has difficulty ejaculating or has severe oligospermia or azoospermia, testicular biopsy should be performed, and the mature or immature testis protocol should be selected depending on the presence or absence of sperm.^27^ Testicular biopsy for cancer patients is done to look for sperm in the testes. The method is the same as the testicular sperm extraction (TESE) performed on azoospermia patients and is called onco‐TESE. The sperm retrieval rate is about 50%–60%.^28^ Patients who have never masturbated are recommended to try using a vibrator or devices, such as those facilitating electric ejaculation, before proceeding to testicular biopsy.^29^ It has been reported that 13.9%–53.3% of adolescent male cancer patients cannot ejaculate.^30^, ^31^ Therefore, it is also necessary to provide guidance to patients and their parents in advance to proceed smoothly with treatment.^32^

Spermatogenesis is not yet complete in prepubertal patients; therefore, sperm are not present in the tissue. Spermatogonia (spermatogonial stem cells [SSC]) collected by testicular biopsy are cryopreserved, and we hope that future technological advances will make it possible to culture sperm from SSCs.^27^, ^33^

### 3.2 Promoting fertility preservation

Widespread awareness of fertility preservation (FP) is very important for the future of cancer survivors. In the United States, 51% of young male cancer patients and 25% of childhood cancer patients want to be fathers in the future.^34^, ^35^ A Japanese study found that one‐third of male cancer patients were worried about fertility, and two‐thirds of patients who underwent FP said working to preserve their fertility gave them the courage to fight cancer.^36^ The ASCO guidelines state that “healthcare providers caring for adult and pediatric patients with cancer should address the possibility of fertility as early as possible before treatment starts” and “healthcare providers should refer patients who express an interest in FP to reproductive specialists”.^18^ However, some oncologists and health care providers (HCPs) are unaware of the developments in sperm banking.^37^ If the doctors and health care providers lack knowledge regarding the developments in the related fields, they may not be able to explain them to the patients. Several reviews have reported barriers to FP, but the causes are lack of communication, lack of resources and time to explain, cost, lack of time to perform FP, and the lack of knowledge among doctors and professionals.^38^ Accordingly, it is necessary for the departments responsible for FP to disseminate related information and cooperate with cancer treatment facilities.

## 4 SEXUAL DYSFUNCTION OF CANCER SURVIVORS

Although not as prevalent as the potential decline in fertility after cancer treatment, the decline in sexual function of cancer survivors is also a serious problem. A 2009 study in the United States found that 49% of male cancer survivors had erectile dysfunction (ED) after treatment, and 30% had orgasm or ejaculation problems.^39^, ^40^ Ritenour et al. surveyed the International Index of Erectile Function (IIEF) score of 1622 male cancer survivors and 271 siblings and found that survivors were 2.63 times more likely to have ED than their siblings.^41^ In this study, the incidence of ED in survivors was 4.2%,^41^ similar to the incidence in men below 40 years of age.^42^ In another study, 62% of AYA generation hematological malignancy survivors had sexual dysfunction.^43^ Such problems if left untreated, may lead to loss of communication between the patient and his partner, and the patient may refuse further treatment. Furthermore, the presence of a partner reduces patient mortality^44^; partner support may be critical in cancer treatment. However, sexual problems can be difficult to discuss even for healthy couples.^45^ Another study reported that oncologists and HCPs have no knowledge of FP.^46^

### 4.1 Pathogenesis of sexual dysfunction after cancer treatments

Sexual dysfunction after cancer treatment includes symptoms of ED, ejaculatory disorder, and hypogonadism in men. ED occurs at a high rate after surgery for prostate cancer,^47^ radiation therapy,^48^ and treatment for rectal cancer.^49^ Amputation of the neurovascular bundle performed during radical prostatectomy or total cystectomy and rectal mesenteric incisions for lymph node dissection can cause ED.^50^, ^51^ Radiation damages the vascular endothelium of the corpus cavernosum, arteries, and nerves of the penis.^52^ Okada et al. evaluated the Sexual Health Inventory for Men (SHIM) score of 52 patients after treatment for testicular cancer over time; sixty‐nine percent of the patients had an SHIM score of 17 or less 2 years after treatment; however, investigative logistic analysis indicated that retroperitoneal lymph node dissection (RPLND) was not a contributing factor toward sexual dysfunction.^53^ The frequency of patients with a SHIM score of 17 or less was found to decrease over time after surgery.

In their reports, nerve amputation was unlikely to be the cause of the ED.^53^ ED is also thought to be caused by a change in body image and a loss of self‐confidence as a man.^54^ Other factors that cause ED include radiation of ≥10 Gy and spinal surgery.^41^ Orgasmic abnormalities have also been reported as sequelae after radical prostatectomy with a frequency of approximately 20%–60% in patients approximately 2 years postoperatively. Symptoms include a lack or delay of orgasm and pain during orgasm.^55^

Ejaculation disorder (EjD) is also one of the most common and serious sequelae after cancer treatment in males and is especially common after RPLND in patients with testicular cancer. Koyama et al. investigated 74 testicular cancer survivors with EjDs. Of the fifty patients whose pretreatment ejaculation status was assessed, nine (18%) reported having EjD after treatment. In a multivariate analysis, RPLND was an independent factor for EjD (*p* = 0.042). They also used multivariate analysis to investigate the causative factors in 60 patients who stopped having sexual intercourse after treatment and found that the presence or absence of a partner and EjD were independent factors for the cessation of sexual intercourse (EjD odds ratio 14.8, 95% CI 1.21–181.26).^56^ EjDs after RPLND include retrograde ejaculation (RE) besides failure of emission; Hsaio et al. examined the cause of postoperative EjD in 26 patients and found four patients with RE.^57^ Brachytherapy for prostate cancer may also cause EjD.^58^

Hypogonadism can be caused by cranial radiation or irradiation of the testes and leads to anemia, muscle and bone mass loss, and menopausal symptoms. Damage to the pituitary gland due to cranial radiation leads to impaired gonadotropic secretion, and 2/3 of patients who receive cranial radiation have symptoms of hypogonadism.^59^ Although less sensitive than germ cells, Leydig cells are also damaged by radiation. When the total pelvic radiation dose is 4 Gy or more, molecular changes can occur,^60^ and if the dose exceeds 20 Gy, androgen supplementation might be needed in the future.^61^ In addition, TKI imatinib is said to damage Leydig cells and cause a decrease in testosterone production.^9^ Besides radiation therapy to the brain, immune checkpoint inhibitors, such as nivolumab, may cause pituitary inflammation and may reduce gonadotropin secretion and subsequent testosterone production. That is, it causes hypogonadotropic hypogonadism, which in turn leads to sexual dysfunction and impairment of spermatogenesis.^62^

### 4.2 Measures and treatments for sexual dysfunction in cancer survivors

Prevention and treatment are required under these conditions. A nerve‐sparing surgical approach can be employed in prostate cancer surgery to prevent ED. Bilateral nerve‐preserving therapy is known to better preserve sexual function when compared to unilateral nerve‐preserving and nonpreserving treatments.^63^ However, 47.3%–88% of patients undergoing nerve‐conserving surgery have been reported to develop ED; therefore, the preventive effect is not very high.^64^, ^65^ The ASCO guidelines on the treatment of sexual dysfunction after cancer treatment recommend using a phosphodiesterase‐5 inhibitor (PDE5i) first and foremost for ED. A vacuum erectile device (VED) or penile intracavernosal injection (ICI) is recommended if PDE5i is not effective.^66^ Postoperative PDE5i efficacy depends on the patient age, preoperative ED presence, nerve preservation, and surgeon skill,^67^ and is heavily used in postoperative rehabilitation and should be introduced early.^66^ In addition to treating ED, VEDs are also effective in preventing the postoperative shortening of penile length. Dalkin et al. reported that 97% of patients could maintain the penile length when VED was used in patients after prostatectomy.^68^ ICI is also a useful tool for ED, but the risk of priapism needs to be communicated to patients.^67^ In addition to these treatments, an implant can also be inserted into the corpus cavernosum, although this is highly invasive. Antimicrobial‐coated prostheses have been developed with a very low risk of infection and high patient satisfaction.^69^

Nerve‐sparing surgery is useful for preventing EjD caused by RPLND. It has also been reported that 93% of patients with bilateral nerve preservation and 74% with unilateral preservation maintained ejaculatory function.^70^ Drugs are mainly used to treat EjDs. Oral tricyclic antidepressants effectively treat RE, but it is difficult to treat FOE patients who cannot completely ejaculate.^67^

The ASCO guidelines recommend counseling for patients and their partners.^66^ Cancer treatment and subsequent sexual dysfunction can significantly affect a couple's body image, confidence, values, satisfaction with future life prospects, and self‐assessment criteria, and their mental health may suffer, leading to mental instability.^71^ It is recommended that counseling be started when the cancer is diagnosed and when necessary. Mental counseling is also said to improve a couples' sexual function.^66^

Unfortunately, there are very few specialists and facilities to address these issues. In addition, the problem of sexual function is not deemed important among oncologists. Bober et al. interviewed 25 National Cancer Institute and National Cancer Comprehensive Network‐registered cancer treatment institutions regarding sexual assistance. Eighty‐two percent of institutions did not have sexual function support for male cancer survivors. On the other hand, 72% of the facilities did not have support for female cancer survivors. Facilities that could not support patients were encouraged to use Google for patients seeking sexual support and introduced URLs of a website and medical device stores specializing in sexual dysfunction support.^72^

## 5 CONCLUSION AND FUTURE DIRECTIONS

The concept of FP is spreading worldwide and is critical to addressing the desires of affected patients. However, FP has not been fully understood and accepted by patients, parents, and guardians, and even HCPs, as an option for fertility after cancer treatment. Considering its straightforward methodology and significant benefits to the patient's quality‐of‐life, it is essential that HCPs learn and disseminate information about FP as an option for patients concerned about their fertility after cancer therapy. Although it is a time‐consuming process, increased use of FP has the potential to provide hope for raising children while facing their incredibly difficult journey as patients with cancer. In addition, for successful FP among affected children, research should aim to accomplish spermatogenesis outside the testis.

Improving sexual function greatly affects the cancer prognosis and postoperative quality of life. Further, FP is a common problem for young cancer survivors. Urologists can treat ED, EjD, and hypogonadism to some extent, but many patients, oncologists, HCPs, and support professionals often do not have access to sufficient information. For young male cancer survivors to lead a “normal” life after treatment, it is important to disseminate knowledge about sexual dysfunction and improve the response to post‐treatment sexual disorders.

## CONFLICT OF INTEREST

The authors declare no conflict of interest.

## ETHICAL APPROVAL

This research was supported by the Ethics Committee of Yokohama City University Medical Center.

## HUMAN/ANIMAL RIGHTS STATEMENTS AND INFORMED CONSENT

This article does not contain any studies with human and animal subjects performed by any of the authors.
